# Deep-Learning-Based Context-Aware Multi-Level Information Fusion Systems for Indoor Mobile Robots Safe Navigation

**DOI:** 10.3390/s23042337

**Published:** 2023-02-20

**Authors:** Yin Jia, Balakrishnan Ramalingam, Rajesh Elara Mohan, Zhenyuan Yang, Zimou Zeng, Prabakaran Veerajagadheswar

**Affiliations:** Engineering Product Development Pillar, Singapore University of Technology and Design (SUTD), Singapore 487372, Singapore

**Keywords:** autonomous mobile robot, environment recognition, DCNN, image classification, contextual features, supervised learning, hazardous object detection

## Abstract

Hazardous object detection (escalators, stairs, glass doors, etc.) and avoidance are critical functional safety modules for autonomous mobile cleaning robots. Conventional object detectors have less accuracy for detecting low-feature hazardous objects and have miss detection, and the false classification ratio is high when the object is under occlusion. Miss detection or false classification of hazardous objects poses an operational safety issue for mobile robots. This work presents a deep-learning-based context-aware multi-level information fusion framework for autonomous mobile cleaning robots to detect and avoid hazardous objects with a higher confidence level, even if the object is under occlusion. First, the image-level-contextual-encoding module was proposed and incorporated with the Faster RCNN ResNet 50 object detector model to improve the low-featured and occluded hazardous object detection in an indoor environment. Further, a safe-distance-estimation function was proposed to avoid hazardous objects. It computes the distance of the hazardous object from the robot’s position and steers the robot into a safer zone using detection results and object depth data. The proposed framework was trained with a custom image dataset using fine-tuning techniques and tested in real-time with an in-house-developed mobile cleaning robot, BELUGA. The experimental results show that the proposed algorithm detected the low-featured and occluded hazardous object with a higher confidence level than the conventional object detector and scored an average detection accuracy of 88.71%.

## 1. Introduction

Over the last two decades, Autonomous Mobile Cleaning Robots (AMCR) have been promising and viable assistive technologies in the cleaning industry. Chen et al. [1] mentioned that there is a high demand for mobile cleaning robots in commercial and industrial applications such as the floor, wall, and table cleaning tasks and they involve a cleaning audit service [2,3]. Hazardous object detection and avoidance are critical functions for autonomous mobile cleaning robots that work alongside humans. Generally, the commercial and industrial sectors are more dynamic; robots and people interact with each other at a high frequency. Moreover, it could be furnished with many objects, and some object robots cannot properly recognize by the perception system. Generally, a staircase, escalator, glass door, and transparent objects are hazardous to mobile robots and need an advanced perception system to detect and avoid these objects under occlusion.

Currently, AMCR’s navigation system widely utilizes 2D LiDAR and 1D laser sensors [4,5], IMU, and Position Sensitive Detectors PSD [6,7] for environment recognition and obstacle detection. However, these sensors’ performances are stable in static environments and relatively weak in accurately recognizing hazardous objects. Moreover, mapping dynamic, highly reflective, or opaque environments using 2D LiDAR can result in incomplete or inaccurate maps [8]. As a result, it could pose a safety issue and cause the robot to make wrong decisions or act incorrectly in autonomously navigating. Recently [9], a shopping mall’s cleaning robot fell from an escalator and slightly injured travellers. This might happen due to the robot’s localization system being affected in dynamic environments or miss detection of the escalator. This incident illustrates that AMCRs need an advanced perception system with functional safety features to accurately detect and avoid hazardous objects.

Object detection using computer vision has been extensively researched for autonomous mobile robot platforms [10,11,12,13]. These methods are cost-effective and can operate in a wide range of scenarios. In recent years, deep-learning-based place recognition, scene recognition, and object detection have been a new paradigm in computer vision techniques and widely used in mobile robot platforms to recognize the environment and avoid obstacles [14,15,16]. Generally, Single Shot multibox Detector (SSD) MobileNet, You only look once (YOLO) and Faster RCNN are widely used deep-learning-based object detectors in mobile robotic applications. In contrast with SSD MobileNet and YOLO, Faster RCNN is widely used for high-precision and safety-critical mobile robot applications. However, Faster RCNN is also weak for detecting low-feature or occluded objects and has miss detection and false classification [17]. Generally, the indoor environment is more challenging than outdoor object detection due to severe occlusions of objects, objects with fewer features, and cluttered backgrounds. In a number of small proposals extracted from the environment, the features computed from a small fraction of the feature map may not be sufficient to accurately determine the object class or provide a high level of confidence in the object detector’s predictions. Furthermore, the object with less confidence can be suppressed by Non-Max Suppression (NMS) algorithms and thus lead the miss-detection of things. Consequently, it creates serious safety issues for mobile robots. One approach to addressing this issue is adding contextual information with a Faster RCNN object detector. Generally, image-level contextual details are more beneficial for visual recognition and object detection applications, mainly when the object of interest is small, blurred, partially occluded, etc. Image-level contextual information provides the semantics of the entire image. Fusing the image-level contextual information with the object proposal feature map will improve the detection algorithms’ classification and regression function and boost the object detector’s confidence level.

This work proposes a deep-learning-based context-aware multi-level information fusion framework for an indoor mobile robot to detect and avoid hazardous object detection in its operational environment. First, the image-level-contextual-encoding module was proposed and incorporated with the Faster RCNN ResNet 50 object detector model to improve the performance of hazardous object detection. Then, a safe-distance-estimation function was proposed. It performs the depth data fusion with detection results to compute the distance of the hazardous object from the robot’s position and steer the robot to a safe zone.

The rest of the article is structured as follows: Section 2 describes the detailed literature survey about related work. Section 3 presents the architecture of the proposed system. The algorithm and experiments are explained in Section 4. Finally, Section 5 concludes the results and future works.

## 2. Related Work

Understanding and exploiting context information is a fundamental problem in computer vision, which has been explored extensively and plays a vital role in many fields [18,19,20,21,22]. Jurang et al. [23] proposed a context-aware co-supervision method to improve the performance of the object detection algorithm, Faster RCNN. The authors developed the context-aware module to assist the Faster RCNN object detection head, which fuses the high-level contextual information with a low-level feature map to detect tiny objects from an input image accurately. The fully convolutional architecture was proposed by Kevin et al. to improve the DL-based object detector’s performance [24]. The author modifies the two-stage DCNN architecture, where the first stage extracts the feature map from the image. The second stage had to learn the local contextual information from the feature map and perform the object detection task. In [25], Zhao et al. developed context-aware deep neural networks for visual content recognition. The author generated the Semantic Feature Map (SFM) by extracting the high-level semantic object features on the input image and applying the Fully Convolutional Networks (FCN) on top of SFM for better visual content recognition.

Raphael et al. [26] proposed a context-aware visual navigation approach for an autonomous mobile robot to find the occluded object in an indoor environment using Yolo v3 and the deep RL algorithm. Here, the authors constructed the context grid from object detection results and then applied the RL algorithm on the context grid to learn the contextual relation between objects. In another study, Luo et al. [27] proposed a contextual-YOLOV3 framework to detect small objects from an input image. The framework builds a contextual relationship matrix and combines contextual semantic information for detecting small objects more accurately. In [28], Ayub et al. proposed a cognitively inspired computational system for an autonomous mobile robot to predict missing items from the household. The system was composed of perceptual learning algorithms and cognitive models of memory encoding to learn the contextual relationship between household environment and use that knowledge to predict missing items from the household. In [29], Li et al. proposed a Cross-Modal Attentional Context (CMAC) method to improve the performance of a region-based object detection framework. Here, the authors used an attention-based context encoding function and a fine-grained object part attention function to extract both global and local feature and fuse it with a region-based object detection feature map to improve the model’s performance and proved 5% improvement over conventional object detectors. Many studies have used context-aware and depth-based fusion to improve object detection, and environment recognition [30]. Li et al. [31] introduced an adaptive fuzzy control algorithm with a 3D mapping guidance system for the underactuated surface vehicle (USV) and unmanned aerial vehicle (UAV). Here, the 3D mapping guidance system provides the reference signals of the yaw degree of freedom for the USV and UAV, and the adaptive fuzzy control algorithm provides position and attitude information by fusing the dynamic surface control (DSC) and the backstepping techniques.

Yu et al. [32] proposed a multi-level information fusion framework to build the robust Vision-Based Simultaneous Localization and Mapping (SLAM) framework. The author used different segmentation methods to extract high and low-level features to facilitate robust localization. The proposed system was tested with real-time driving datasets, scoring better robustness and consistency than the SOTA schemes. The Soldier-Body Sensor Network (S-BSN) was proposed by Han et al. [33], where the network collects the different types of data such as behaviours, physiology, emotions, fatigue, environments, and locations using wearable body sensors and performs the multi-level fusion to analyze and alerts the soldier’s health when involved in extreme events. Wang et al. proposed context-aware compositional nets for detecting an object on different levels of occlusions [17]. The author segmented the contextual information via bounding box annotations and used the segmented information to train the context-aware CompositionalNet. The trained model has been validated with PASCAL3D+ and MS-COCO datasets and scored 41% improved detection accuracy than the conventional scheme. Abid and Tahir proposed the multi-sensor fusion-based mobile robot fault detection and isolation (FDI) method [34] where the authors incorporate preprocessing, local-data fusion, change detection, credibility computation, and decision-level information fusion to assist the robot in navigation and fault detection. Saeedi et al. proposed context-aware multi-sensor data fusion algorithms that include preprocessing, feature detection, feature selection, and classification to improve the accuracy and robustness of the Personal Navigation System (PNS). The authors proved that the context-aware sensor fusion scheme had improved the performance of PNS by 23% compared to conventional GPS-based navigation [35]. The above survey indicates that deep-learning-based context-aware multi-sensor fusion systems can enhance the functional safety of robots by providing them with a deeper understanding of their surrounding environments. However, context-aware vision pipelines for mobile robots’ hazardous object detection still need to be studied. Hence, this study proposed a context-aware, multi-level information fusion system for indoor mobile robot’s hazardous object detection application.

## 3. Proposed System

**Figure 1:**
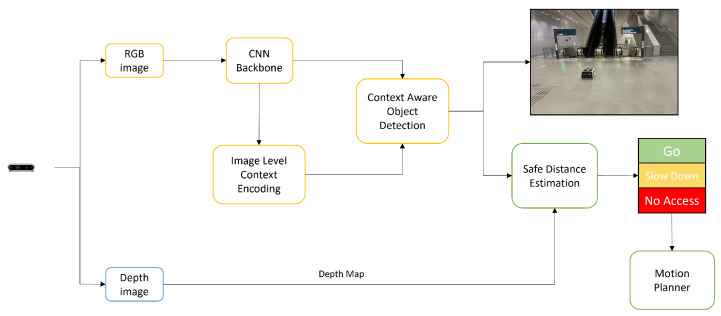
Block diagram of proposed system.

Figure 1 shows the block diagram of deep learning (DL)-based context-aware multi-level information fusion systems for indoor mobile robots’ hazardous object detection and avoidance. The framework comprises context-aware DCNN-based object detection algorithms and a safe-distance-estimation function.

### 3.1. Context Aware DCNN-Based Object Detection

**Figure 2:**
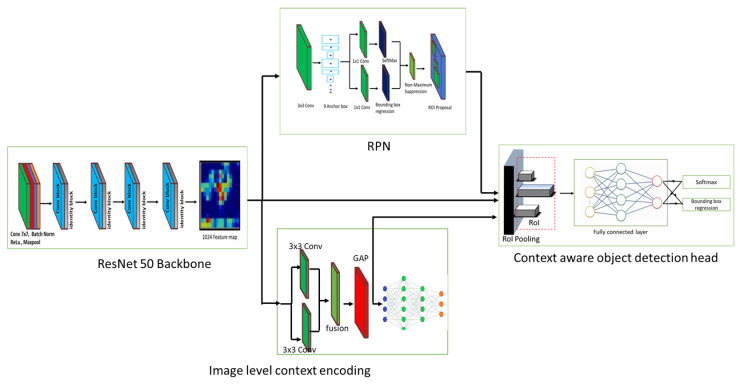
Context aware DCNN-based object detection framework.

In this paper, we incorporated an image-level-contextual-encoding module to the two-stage object detector Faster RCNN to build the context-aware object detector as shown in Figure 2. It comprises a backbone network, a Regional Proposal Network (RPN), an image-level context encoding module, and a context-aware object detection head.

#### 3.1.1. Backbone Network

ResNet 50 Deep Neural Network (DNN) was used as a backbone for our proposed system. It contains 48 convolutions layers and one max pooling and average pooling layer. The first layer includes 64, 7 × 7 kernel convolution with the stride of 2 and 3 × 3 max pooling function with a stride of 2. The next four stages are made up of a mix of residual convolution blocks, and identity blocks contain (1×1, 3×3, 1×1) convolution filters with different counts. The backbone network extracts the feature map from the image and serves to image-level context encoding module, the Region Proposal Network (RPN), and the context-aware object detection head.

#### 3.1.2. Region Proposal Network

RPN is an FCN (Fully Convolutional Network) trained end-to-end to produce object proposals. It uses the backbone-generated feature map as input and creates object proposals using fixed-size anchor boxes. RPN uses nine different size & ratio anchor boxes and applies a 3 × 3 sliding window function to detect the object in the feature map. After that, each object proposal is assigned a score, with the highest-scoring proposals given the highest priority. This ranking helps to ensure that the most promising proposals are examined first, which can help to save time and computational resources. Besides that, the object proposal with a high degree of overlap is considered redundant and removed by NMS. After the object proposals are scored and ranked, they are fused with the last convolutional feature map of the backbone network. In RoI pooling, each feature region in each object proposal is max-pooled into a regional object feature map with a dimension of 7 × 7 × 512.

### 3.2. Image-Level Context Encoding Module

The image-level context encoding module constructs the global feature map from the backbone-generated feature map. Then, it fuses it with the RPN-generated feature map to bring the clues to the detection head. First, it applies an encoding operation using two parallel dilated convolutional layers with 512 convolutions filters to capture object classes appearing in the entire image. Then, a global average pooling operation is applied to the encoded feature map and sent to feature map fusion and object detection tasks.

### 3.3. Context Aware Object Detection Head

The context-aware object detection head determines the category of the object contained within each proposal by utilizing an image-level context encoding module 7 × 7 × 512 feature map as well as an RPN-generated 7 × 7 × 512 feature map. In the initial stage, feature map concatenation is performed, where an RPN-generated feature map is fused with an image-level contextual feature map. This process enlarges the feature map depth to 7 × 7 × 1024 dimensions. Then, the concatenated feature map is fed into the object recognition and bounding box refinement module, which detects and classifies the bounding box of the predicted objects in the image. In the end, NMS is applied to eliminate redundant bounding boxes.

### 3.4. Safe-Distance-Estimation Function

The safe-distance-estimation function module measures the distance of the hazardous object from the robot’s current position using depth data collected from RGB-D vision sensor data. First, the function takes the bounding box coordinates of the detected object from the RGB image and fetches the respective depth data for each bounding box from the depth image. Then, Realsense rs-measure API [36] was applied on selected depth regions which measured real-world distances of the object from depth data. In the end, the measured object distance was sent to the robot control. This will aid the robots in recognizing whether they are operating in a safe zone or close to hazardous regions.

## 4. Experiments and Results

This section evaluates the effectiveness of the context-aware multilevel information fusion framework at three levels: dataset preparation and training, validation with test image datasets, and experimentation with our in-house cleaning audit robot platform, BELUGA [3].

### 4.1. Dataset Preparation

Our dataset contains seven potential hazardous objects, including escalators, moving walkalator, elevators, glass doors, staircases, glass-made display cabinets, and modern furniture. The hazardous objects are labeled with the bounding box annotation tool and have 1200 samples for each class. Furthermore, data augmentation (rotation, scaling, and flipping) is applied to all collected images to reduce the CNN learning rate and avoid over-fitting.

### 4.2. Training Hardware and Software Details

The context-aware object detection algorithm was developed in TensorFlow 2.13 API and trained using the Nvidia Geforce GTX GPU-enabled workstation. The entire DCNN network was trained using the fine-tuning method in three phases. In the first phase, the backbone, RPN, and context-aware object detection head were fine-tuned with image-Net pre-trained weights for object proposal generation and to detect the objects from the RPN proposals. The loss function of RPN is given in the equations (Equations (1) and (2)). It was the sum of classification loss and regression loss. The binary cross-entropy loss was used to compute loss over the two classes (whether it is an object or background). Further, L2 regression loss was used to compute the bounding box offset, which computes the difference between the regression of the foreground box and that of the ground truth box. Finally, multi-class cross-entropy loss (Equation (3)) was used in the context-aware object detection head, which computes the multi-class classification loss for each training example. Further, the global context encoding module CNN layers were fine-tuned with ImageNet pre-trained weights using a separate ResNet 50 backbone. In the third phase again, the global context encoding module (initialized from fine-tuning weights of stage 1) and its fully connected layer were fine-tuned with the stage 1 fine-tuned backbone network to learn more features about the targeted object feature. In this phase, the layers of backbone architecture were kept frozen to prevent the update of weights during the backpropagation. Under the fine-tune method, the detection model was trained with 80 k epochs using the stochastic gradient descent method, a batch size of 2, an initial learning rate of 0.004, momentum of 0.9, and weight decay of 0.00001, respectively.

(1) $$L\left(\left\{p_{i}\right\},\left\{t_{i}\right\}\right)=\frac{1}{N_{cls}}\sum_{i}L_{cls}\left(p_{i},p_{i}^{*}\right)+\lambda \frac{1}{N_{reg}}\sum_{i}p_{i}^{*}L_{reg}\left(t_{i},t_{i}^{*}\right)$$

(2) $$L=L_{cls}+L_{reg}$$

In Equations (1) and (2), *i* is the index of anchor, *p* is the probability of an object or not, *t* is the vector of four parameterized coordinates of the predicted bounding box, and * represents ground truth box. $L_{cls}$ represents Log Loss over all classes. $N_{cls}$ and $N_{reg}$ are normalization. λ defaults to 10 and it is to scale with the classifier and regressor on the same level.

(3) $$L\left(\hat{y},y\right)=-\sum_{k}^{K}y^{\left(k\right)}\log_{}{\hat{y}}^{\left(k\right)}$$

In Equation (3), $y^{\left(k\right)}$ is 0 or 1, indicating whether class label *k* is the correct classification.

### 4.3. Prediction of Hazardous Object Detection

**Figure 3:**
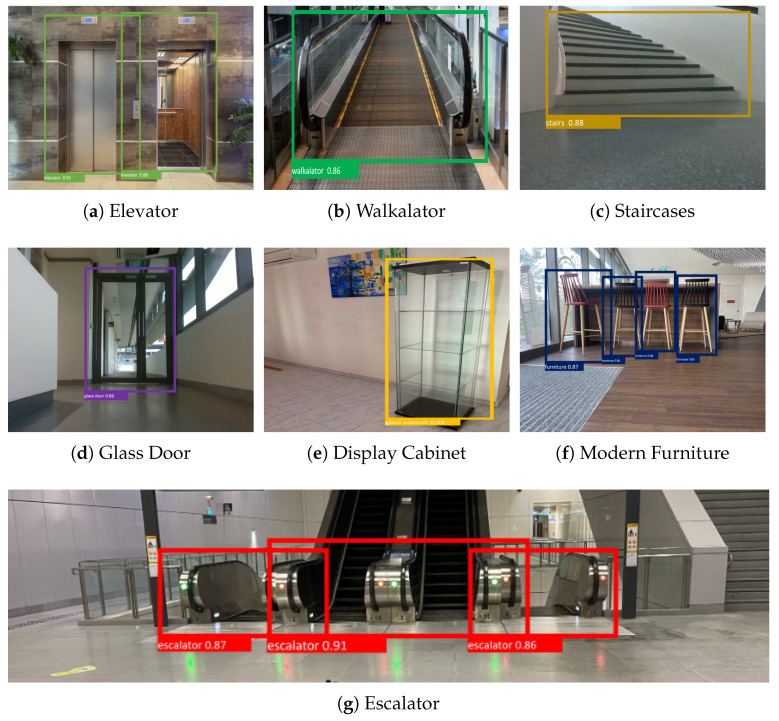
Experiment results of hazardous object detection.

Figure 3 shows the prediction results of our proposed system. In total, 200 images were used for each class to measure the performance of the trained model. The image was not used for training and cross-validation of the model. *Accuracy* (Equation (4)), *Precision* (Equation (5)), *Recall* (Equation (6)), and $F_{measure}$ (Equation (7)) IoU (Intersection over Union) metrics were used to measure the performance of the proposed scheme.

(4) $$Accuracy\left(Acc\right)=\frac{tp+tn}{tp+fp+tn+fn}$$

(5) $$Precision\left(Prec\right)=\frac{tp}{tp+fp}$$

(6) $$Recall\left(Rec\right)=\frac{tp}{tp+fn}$$

(7) $$F_{measure}\left(F_{1}\right)=\frac{2\times precision\times recall}{precision+recall}$$

Here, tp,fp,tn,fn represent the true positives, false positives, true negatives, and false negatives, respectively, as per the standard confusion matrix.

The experimental results (Figure 3 and Table 1) indicate that our proposed system detects a hazardous object with an average confidence level of 87%, classification error (localized correctly but classified incorrectly) of 8% on average, a mean IoU score of 0.77, and an average detection accuracy of 88.71%.

### 4.4. Comparison Analysis with Conventional Method

**Figure 4:**
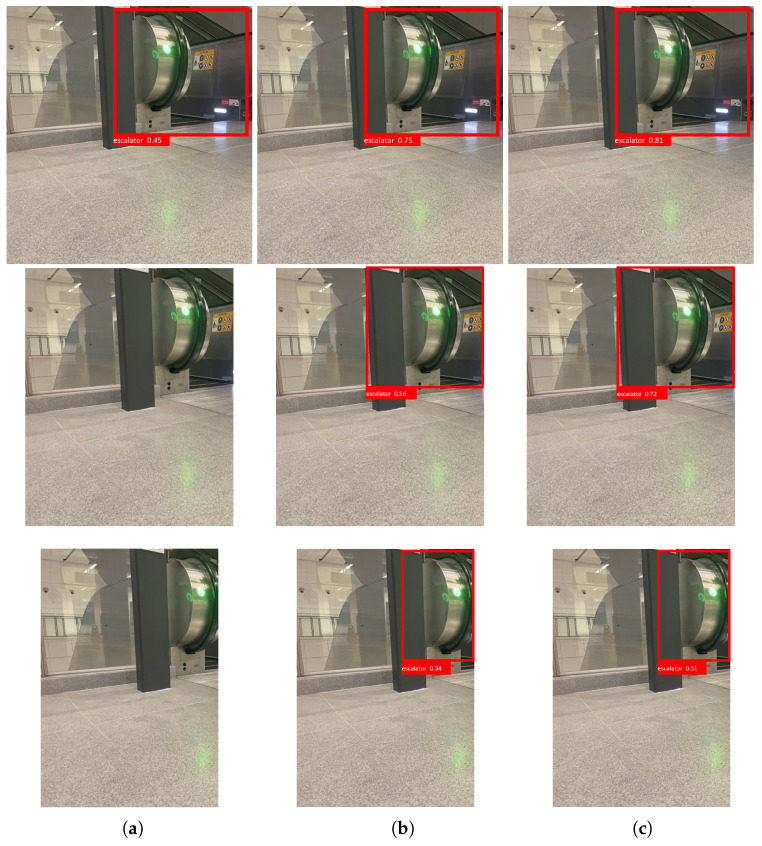
Comparison analysis results of the context-aware object detection algorithm and conventional object detection scheme for the escalator and glass door: (**a**) Yolo V4; (**b**) Faster RCNN ResNet 50; (**c**) Proposed system. From top to bottom, the occlusion conditions are shown from low, medium, and high levels.

The effectiveness of the proposed system was compared with the state-of-the-art object detection methods, including Faster RCNN ResNet 50 and Yolo V4. Figure 4 and Table 2 show the proposed system’s comparative analysis and computational time with state-of-the-art object detection methods. Each algorithm’s computational time was estimated using the number of images processed per second. The experiment was tested using the Nvidia Jetson AGX Xavier single-board computer powered with 512 NVIDIA CUDA cores, 64 Tensor cores, and two DL accelerators.

Figure 4 shows the conventional methods and proposed method detection results for a hazardous object under different occlusion conditions, such as low, medium, and high levels. In this analysis, we observe that the single-stage object detector Yolo v4 has failed to detect the mid- and high-occluded hazardous objects. On the other hand, Faster RCNN ResNet 50 detects the mid-level-occluded hazardous objects with a lower confidence level and fails to detect a high-level-occluded hazardous object. In contrast with Yolo v4 and Faster RCNN ResNet 50, our proposed method detects the low- and mid-level hazardous object with a higher confidence level and detects the highly occluded hazardous object with an average confidence level of 85%. Further, the detection accuracy analysis (Table 2) shows that our proposed method shows 6.38% improved detection accuracy than the baseline method Faster RCNN ResNet 50 and 13.85% higher detection accuracy than Yolo V4 algorithms, respectively.

From a computational point of view, the YOLOv4 framework took less execution time than all other models. Our method has a higher computational time and processes only four images per second, slightly lower than the conventional method. Due to image-level-encoding fusion, our scheme consumes more computation time than the other two methods.

### 4.5. Performance Analysis Survey

Table 3 shows the performance analysis of our proposed work with existing similar works in the literature. Here, staircase detection was compared with Unmesh et al. [37], Wang et al. [38], and Afif et al. [39] methods where the authors use the tiny-Yolo V2, SE-ResNet, YoLov5, and Yolo V3 framework for detecting the staircase. Further, glass door detection was compared with Mei et al. [40] and Afif et al. [39] schemes, where the author uses pre-trained ResNet101 and Yolo V3 to extract the contextual features at different levels to detect the glass door from the RGB image. Elevator and furniture detection was compared with Afif et al. [39] and Alejandra et al. [41] schemes. Here, Yolo V3 and SVM algorithms were trained for the elevator and furniture detection tasks.

The performance analysis results indicate that our proposed method scored better detection accuracy than existing methods. Furthermore, our approach has less miss detection and false classification due to the fusion of global contextual information with object proposal. Therefore, it could increase detection accuracy compared to existing schemes.

### 4.6. Real-Time Field Trial with Safe Distance Estimation

**Figure 5:**
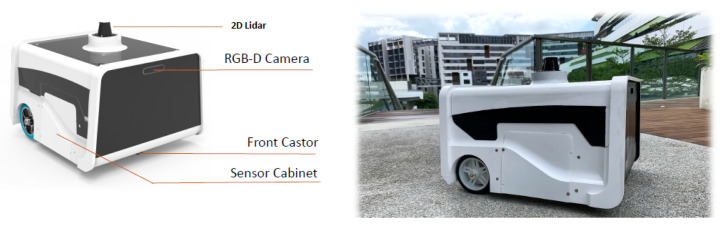
Experiment Robot [3].

**Figure 6:**
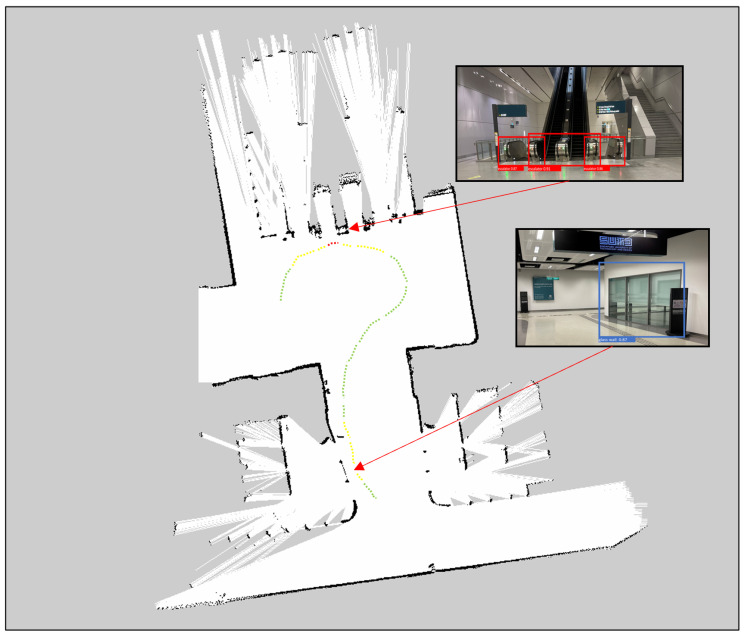
Environment: SUTD Mass Rapid Transition (MRT) station.

**Figure 7:**
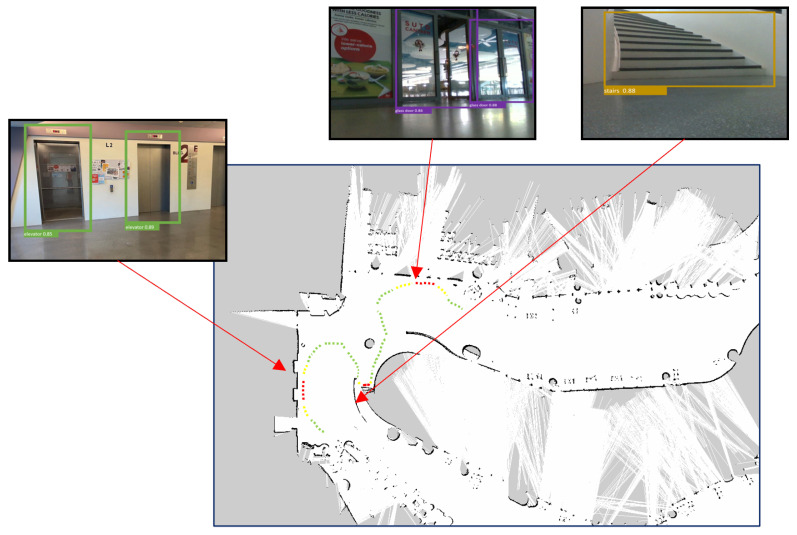
Environment: SUTD campus.

The real-time field trial experiments were performed in our Singapore University of Technology and Design campus using BELUGA (Figure 5). The detailed specification of the robot is given in Table 4. Initially, the environment was mapped with 2D lidar using hector SLAM algorithms. After mapping, the mapped environment was tested with BELUGA [3]. The context-aware object detection algorithm and safe-distance-estimation function were run on Jetson Nvidia AGX SBC to detect the hazardous object in an operational environment. Whenever a hazardous object detected an input image, the detection results were forwarded to compute the distance of the object from the robot’s position. The distance estimation function took the bounding box coordinates of the detected object from the RGB image and fetched the respective depth data for each bounding box from the depth image. Then, Realsense rs-measure API [36] was applied on the selected depth regions which measured real-world distances of the object from depth data. In the end, the estimated object distance was sent to the robot control. Figure 6 and Figure 7 shows the robot navigation path for the given environment with functional safety mapping results when the robot navigates the staircase, elevator, and glass door region. In Figure 6 and Figure 7, the green, yellow, and red colored dotted lines indicate the navigation path for the robot. The green navigation path represents the robot operating in a safe zone. The yellow navigation path represents that the robot is approaching hazardous objects between 0.5 m to 1 m and regenerates a new path to avoid them. The red color navigation path represents the robot close to the hazardous object (less than 0.5 m) and should immediately turn to a safe zone.

## 5. Conclusions

A deep-learning-based context-aware multi-level information fusion framework was proposed for autonomous mobile cleaning robots to detect and avoid hazardous object detection in their operational environment. First, an image-level context encoding module was proposed. Its feature map results were fused with Faster RCNN region proposals to improve the hazardous object detection confidence level and detect the hazardous object on a different level of occlusions. Then, depth data fusion was performed with detection results to compute the distance of the hazardous objects from the robot position. The proposed framework was trained with a custom dataset using a fine-tuning method. Its detection accuracy was evaluated offline with a test image dataset and in real-time using the cleaning audit robot, BELUGA. In our offline test, our proposed scheme scored an average of 88.71% detection accuracy. It processed four images per second and detected the occluded hazardous objects with a higher confidence level than conventional methods such as Faster RCNN and Yolo V4. Compared with existing works, our proposed method scored better detection accuracy for staircase, elevator, glassdoor, and furniture classes. Further, the safe distance estimation map results ensure that our proposed system accurately computed the distance of the hazardous object, which helps steer the robot to a safe zone. Our feature work is the local and global context feature-map-based hazardous object detection for the safe navigation of indoor mobile robots. It could further increase the object detector performance and improve the operational safety of our autonomous mobile cleaning robot.

## Figures and Tables

**Table 1:** Performance Evaluation.

Class	Proposed System			
	Precision	Recall	$F_{1}$	Accuracy
Elevator	90.35	89.76	87.52	87.76
Escalators	89.84	89.11	88.66	89.18
Walklator	89.76	88.17	86.09	89.33
Glass door	88.54	87.25	87.37	87.22
Staircase	93.51	92.44	91.03	91.77
Display cabinet	86.01	85.31	84.76	85.43
Modern furniture	87.61	86.29	86.18	88.78

**Table 2:** Comparison with conventional methods.

Algorithm	Detection Accuracy	Number of Image Processed per Second
Yolo V4	74.86	23
Faster RCNN ResNet 50	82.33	9
Proposed system	88.71	4

**Table 3:** Comparison with other defect detection schemes.

Case Study	Algorithm	Detection Accuracy in (%)
Staircase [37]	Yolo V2 CNN	77.00
Staircase [38]	SE-ResNet	81.49
Staircase [38]	YoLov5 + Gabor	37.3
Staircase [39]	Yolo V3	76.88
Glass door [39]	Yolo V3	85.55
Glass door [40]	ResNet101	81.63
Elevator [39]	Yolo V3	85.04
Furniture [41]	SVM	71.45
Proposed system	Faster RCNN+ image level encoding	88.71

**Table 4:** BELUGA Specification.

Components	Details
RGB-D Camera	Intel Realsense 435i
On-Board IDK	NVIDIA’s Jetson AGX GPU
2D LIDAR	Sick TIM 581
Power	24VDC LiFePO4 battery powers

## Data Availability

Data will be shared based on request.
